# New Right‐To‐Left Shunt Through a Patent Foramen Ovale Following Abdominal Surgery

**DOI:** 10.1002/ccr3.71713

**Published:** 2026-03-12

**Authors:** Jack Franke, Christopher Koo, John D. Serfas, Matea Malinovic, John Ashcraft, Brigid C. Flynn

**Affiliations:** ^1^ University of Kansas Medical Center Kansas City Kansas USA; ^2^ Department of Surgery University of Kansas Medical Center Kansas City Kansas USA; ^3^ Department of Cardiology University of Kansas Medical Center Kansas City Kansas USA; ^4^ Department of Anesthesiology University of Kansas Medical Center Kansas City Kansas USA

**Keywords:** echocardiography, patent foramen ovale, platypnea orthodeoxia, right‐to‐left shunt

## Abstract

In the setting of new platypnea‐orthodeoxia syndrome, early consideration of right‐to‐left shunting via a patent foramen ovale is critical for effective management.

## 1 Introduction

We present the case of a new‐onset, hemodynamically significant right‐to‐left shunt through a previously unrecognized patent foramen ovale (PFO) following intra‐abdominal tumor resection complicated by postoperative increased intra‐abdominal pressure. This condition manifested with platypnea‐orthodeoxia syndrome, which was unresponsive to oxygen therapy. Echocardiographic and CT imaging demonstrated a conformational change in the right atrium, leading to the development of the right‐to‐left shunt via the PFO. Subsequent percutaneous closure of the PFO led to resolution of both the shunt and the symptoms of platypnea‐orthodeoxia.

## 2 Case History

After obtaining patient written consent, we present a 72‐year‐old Caucasian woman with a history of subclinical lacunar infarcts who was found to have a neuroendocrine tumor of the small bowel. She had no cardiopulmonary history or symptoms. She had undergone a stress test 7 years prior and an echocardiogram 6 years prior. Both tests were normal. Since she had no further cardiopulmonary symptoms with an active lifestyle, she was deemed safe to proceed for surgery.

She underwent open ileocecetomy with primary anastomosis. During the first postoperative night, she had significant abdominal distention, pain, and hypotension requiring intravenous norepinephrine and vasopressin support. She also developed worsening hypoxemia with oxygen saturation (SaO2) of 80% despite administration of 60 L of high flow 100% oxygen. Her mental status remained normal. She also became anuric despite an attempt at diuresis with rising serum creatinine, possibly due to intraabdominal hypertension. Her abdomen was distended, which was felt to be due to postoperative changes and edema. However, her ongoing severe hypoxemia was deemed unrelated and to be associated with a larger risk of mortality than her potential intraabdominal hypertension; thus, she was not taken for exploratory laparotomy at this time.

Notably, her hypotension and hypoxemia were worse when the patient was sitting upright and improved when the patient was laid flat or in Trendelenburg position, consistent with platypnea‐orthodeoxia syndrome. Additionally, administration of crystalloid boluses (1–2 L at a time) rapidly corrected her hypoxemia. Also, during fluid administration, her hypotension improved, allowing weaning of her vasopressor infusions. However, upon completion of the fluid boluses, her hypoxemia and hypotension worsened again. This finding was not consistent with intraabdominal hypertension. The authors theorize that the fluid boluses created more flow into her right ventricle and subsequently through her pulmonary vasculature, allowing for oxygenated blood to be delivered to her body.

**FIGURE 1:**
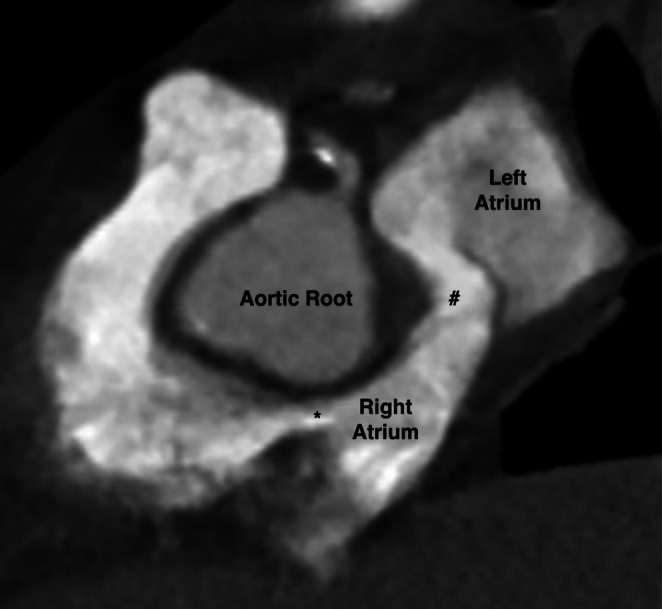
CT image demonstrating the septum primum of the interatrial septum (#) bowing into the left atrium with contrast flowing through the PFO. Note the compression of both the right and left atrium and the tricuspid annulus (*).

**FIGURE 2:**
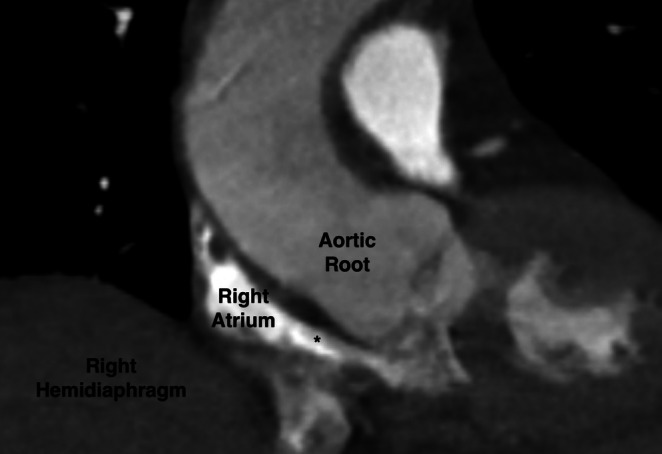
CT scan demonstrating the compression of the right atrium against the aortic root due to elevation of the right hemidiaphragm.

On postoperative day (POD) 1, her chest X‐ray was unremarkable. A CT angiogram of the chest was performed to exclude pulmonary embolism. It was negative for pulmonary embolism, pulmonary edema, or pneumonia but did demonstrate a significant right‐to‐left bulge of the interatrial septum, which measured 1.1 cm from the normal plane of the septum. This was suspicious for a PFO (Figures 1 and 2). Importantly, the CT also demonstrated elevation of both diaphragms with trace ascites. The right atrium and ventricle were compressed between the elevated diaphragm and a mildly dilated aortic root, which led to the shortening of the interatrial septum.

**FIGURE 3:**
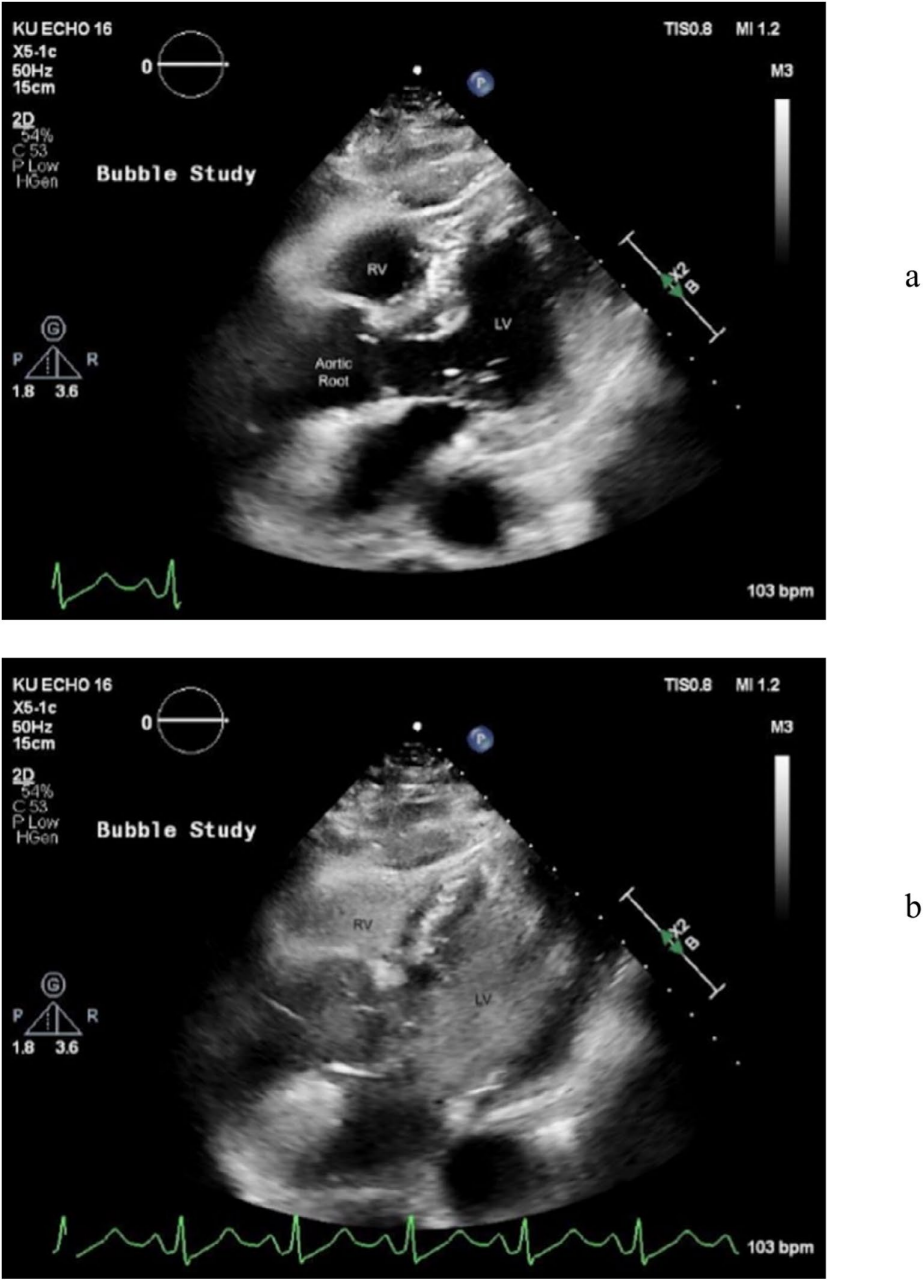
Transthoracic echocardiogram images of pre‐ (a) and post‐ (b) bubble study demonstrating complete and equal opacification of both the right ventricle (RV) and the left ventricle (LV) that make the blood appear speckled white and gray.

Echocardiogram demonstrated normal left ventricular wall motion and ejection fraction of 68%, with a very small right atrium and ventricle, including a very small tricuspid annulus and an interatrial septal aneurysm. The right ventricle was not well seen due to small size. A bubble study demonstrated near simultaneous filling of both the left and right ventricles during agitated saline injection (Video 1). This was diagnostic of a large right‐to‐left intracardiac shunt, with high suspicion for a PFO (Figure 3a,b).

**VIDEO 1:** Saline contrast echocardiogram, or bubble study, demonstrating a right to left shunt across a patent foramen ovale (PFO). The bubbles cross the PFO and the tricuspid valve at approximately the same time allowing for filling of the right ventricle and the left ventricle nearly simultaneously. Video content can be viewed at https://onlinelibrary.wiley.com/doi/10.1002/ccr3.71713.

Her echocardiogram from 6 years prior was reviewed and was unremarkable with normal‐sized cardiac chambers, but no bubble study was performed to evaluate for PFO.

Cardiology was consulted to evaluate the patient for potential PFO closure. On POD 2, she underwent catheter‐directed repair of her PFO with a 25 mm Gore Cardioform device. The decision not to intubate was made from concern that the associated increase in intrathoracic pressure could worsen the right‐to‐left shunt and increase her hypoxia. Thus, she remained on heated high‐flow oxygen.

## 3 Differential Diagnosis

The patient's symptoms initially suggested the possibility of intra‐abdominal hypertension or pulmonary embolism. However, the unresponsiveness to high‐flow oxygen therapy, coupled with the patient's improvement in the supine position, was consistent with platypnea‐orthodeoxia syndrome, which suggested a different etiology. A differential diagnosis included:

– *Pulmonary embolism* in the setting of malignancy and recent surgery.
– *Intrapulmonary shunts* (e.g., pulmonary arteriovenous malformations)
– *Postoperative changes in abdominal and thoracic anatomy* leading to a right‐to‐left shunt via PFO.

Imaging studies (CT angiogram and echocardiogram) ultimately pointed to a right‐to‐left shunt through a PFO, which was confirmed by the bubble study.

## 4 Conclusion and Results

**FIGURE 4:**
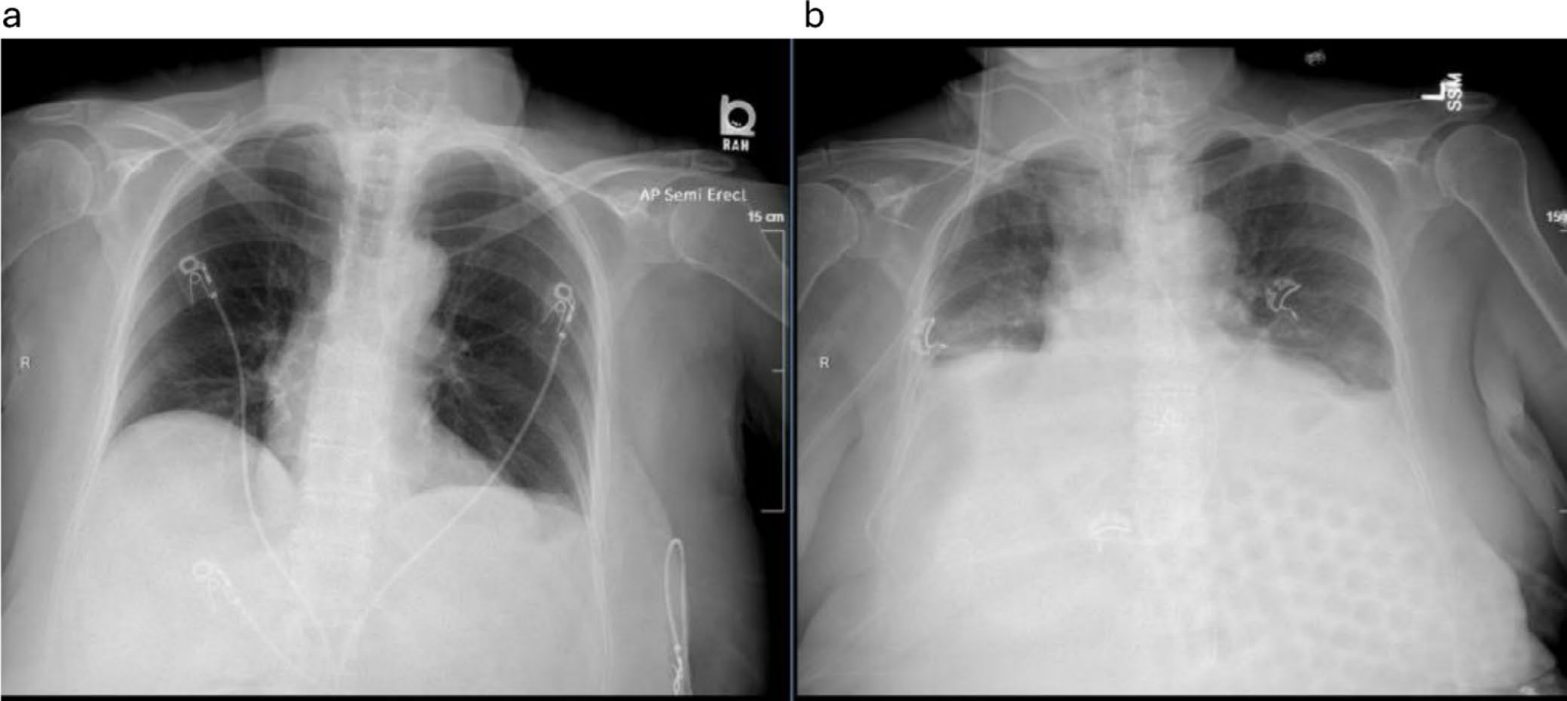
Chest x‐ray demonstrating progression of respiratory failure. (a) postoperative Day 1, (b) postoperative Day 12.

After correction of the right to left shunt, the patient was quickly weaned off supplemental oxygen and vasopressors. Unfortunately, on POD 13, she was found to have new onset hypoxemic and hypercarbic respiratory failure in the setting of a worsening abdominal exam. Her chest x‐ray had worsened with decreased lung volumes, bilateral pleural effusions, and atelectasis (Figure 4). A CT scan of the chest, abdomen, and pelvis revealed superior mesenteric venous thrombosis and small bowel ischemia. She was taken urgently to the operating room and found to have diffuse ischemic bowel and perforation extending from the mid‐jejunum to the splenic flexure. The entire length of necrotic bowel was resected and an end jejunostomy created. The patient continued to be hypoxemic and she, along with her family, decided to pursue comfort measures and she passed away on POD 18. Data available upon request.

## 5 Discussion

An embryonic interatrial connection called the foramen ovale allows shunting of fetal blood in a right‐to‐left direction. In 75% of the population, the foramen ovale spontaneously closes at birth due to an increase in left atrial pressure; however, a PFO persists in 15%–35% of individuals [1]. Platypnea refers to a worsening of dyspnea which occurs in the upright position and improves with recumbency. Orthodeoxia refers to an arterial oxygen tension drop by more than 5% or 4 mmHg in the upright position. The most common causes of platypnea‐orthodeoxia syndrome include intrapulmonary shunts, such as pulmonary arteriovenous malformations, and hepatopulmonary syndrome.

Acute, new onset platypnea‐orthodeoxia syndrome often coincides with a shunt and thoracic or abdominal structural abnormalities [2]. Following intrathoracic or intra‐abdominal surgery, platypnea‐orthodeoxia syndrome can result from a dynamic right‐to‐left cardiac shunt through a PFO induced by anatomical changes in the relative position between the inferior vena cava and the PFO, such that caval blood flow is directed by the Eustachian valve across the PFO, even in the absence of elevated pulmonary and right atrial pressures [3].

When right‐to‐left shunting between the atria causes deoxygenated blood to enter the systemic circulation, the PFO becomes clinically significant. This results in systemic hypoxemia and can be worsened by reduced left ventricular preload or increased afterload of the right ventricle. With Valsalva maneuvers, pulmonary hypertension, positive pressure ventilation, or postoperative anatomical changes, increased right ventricular afterload occurs. These conditions exacerbate the right to left shunt, further diverting blood flow away from the pulmonary vasculature through the path of least resistance, that is, through the PFO and into the left atrium.

In this patient's case, there were increased thoracic pressures directly translated across the diaphragm from increased abdominal pressures due to postoperative abdominal distention. The increased intra‐abdominal and intrathoracic pressures created a conformational change in the thoracic cavity affecting the most compliant and, therefore, vulnerable cardiac structures, the right atrium and ventricle. The interatrial septum became shortened and was squeezed between the aortic root and the upwardly moving diaphragm, allowing the PFO to open. This led to a conformational change, whereby blood from the inferior vena cava was directed at the open PFO. Since the right atrium and right ventricle compliance were decreased due to compression, blood was preferentially shunted across the PFO. Right ventricular preload was further depleted due to attempts of diuresis to improve oxygen saturation, which further worsened the shunt.

Platypnea‐orthodeoxia cases have been reported as the first symptom in diagnosing a new or worsened PFO shunt in surgical patients. Intrathoracic surgical cases which have reported new PFO shunting include following pneumectomy [4] and following single‐lung transplantation [5]. These cases are thought to occur due to the myriad of anatomical and pressure‐related changes in the thoracic cavity after these types of surgeries. These changes all favor right‐to‐left shunting in the presence of PFO.

Intra‐abdominal surgeries have also been reported to create conformational changes in the thoracic cavity as occurred in our patient due to abdominal distention. Other authors have reported a new PFO in a patient who underwent multi‐organ resection for ovarian cancer and had platypnea‐orthodeoxia that was refractory to oxygen therapy [6]. Echocardiography successfully identified the PFO, and subsequent percutaneous closure led to the resolution of platypnea‐orthodeoxia syndrome. Other cases of development of a new PFO formation and shunting involved patients who underwent hepatic resection [7] and Nissen fundoplication [8].

Nonsurgical factors causing platypnea‐orthodeoxia, such as right hemidiaphragm elevation, can lead to direct cardiac conformational changes. One report involved a patient with muscular dystrophy [9] and another in a patient with a large hepatic cyst [10]. In another nonsurgical case, platypnea‐orthodeoxia syndrome was the first sign prompting an investigation in a patient with collapse of the left lower lobe of the lung [11]. The mediastinal shift caused by lung collapse led to new right‐to‐left shunting through the PFO. The authors believed that in an upright position there was stretching and widening of the patient's PFO due to gravity, which resulted in an increased shunt despite normal intracardiac pressures. After relieving the lung collapse with a bronchial stent, the mediastinum returned to its normal position, leading to the resolution of platypnea‐orthodeoxia syndrome. Again, the presenting symptom in these surgical and nonsurgical reports was platypnea‐orthodeoxia, prompting further investigations and identification of the PFO.

To manage a newly diagnosed PFO, efforts should focus on alleviating hypoxemia and optimizing volume status until definitive treatment can be applied. Deciding whether to proceed with endotracheal intubation and initiate positive pressure ventilation should be tailored to each individual patient's situation. The increased intrathoracic pressures with positive pressure ventilation will increase right ventricular afterload and decrease right atrial compliance. Both changes will worsen right to left shunting. Thus, intubation may not improve hypoxemia.

Alternatively, high work of breathing effort, hypercarbia, acidosis, and hypoxia without an endotracheal airway can have numerous negative effects. When respiratory muscles become fatigued, intrathoracic pressure rises, and a significant amount of oxygen is consumed. Hypercarbia and acidosis are both potent pulmonary vasoconstrictors leading to increases in right ventricular afterload and shunting. Importantly, end organs, such as the kidneys and bowels, rely on adequate perfusion and oxygenation. However, there is no guarantee that intubating will improve oxygenation if the shunt remains.

Definitive management of shunting via PFO is correction of the PFO itself. This historically was only possible surgically, but in current practice the vast majority of PFOs can be readily closed percutaneously with a closure device. The Amplatzer PFO Occluder and the Gore Cardioform Septal Occluder are the two most commonly used devices in the United States, both featuring self‐expanding nitinol frames with a dual‐disc design [12]. Femoral venous access is obtained and the delivery system is placed across the PFO. The left atrial disc is formed in the left atrium before being retracted against the interatrial septum, and then the right atrial disc is deployed on the right atrial side of the septum, effectively closing the PFO. Endothelialization of the closure device, which typically takes 3–6 months, is required for complete sealing of the PFO. However, the magnitude of the right‐to‐left shunt is dramatically reduced immediately upon device closure and should resolve almost all clinically significant hypoxia related to right‐to‐left shunting [12].

After placing the device, the patient in this report experienced notable improvements in oxygen saturation and blood pressure. Although the closure device was successful in closing the PFO and the patient had great benefit in terms of respiratory and renal recovery, the 2‐day period of hypoxemia, hypotension and high pressor use likely caused smoldering bowel ischemia which progressed to overt necrosis and perforation. While the identification and correction of the cause of hypoxia in her case was swift, ischemic damage to the bowels following abdominal surgery can happen quickly.

In summary, newly developed right‐to‐left shunting through a PFO following intrathoracic or intra‐abdominal surgery is a rare but important potential cause of postoperative hypoxemic respiratory failure. Suspicion of such a shunt should be heightened if oxygenation changes correlate with platypnea‐orthodeoxia syndrome. Once a shunt diagnosis is established, a detailed understanding of the intrathoracic and intra‐abdominal anatomy and pathophysiology is crucial for effective patient management. Maneuvers to decrease hypoxia may include maintenance of supine position and avoidance of increases in intrathoracic pressures. Endotracheal intubation should be performed with caution and should not be required for most cases unless non‐shunt‐related indications for intubation are present. In the case of hypotension, the administration of intravenous fluids or vasopressors should be individually tailored to each patient's unique physiology. Finally, consultation of interventional cardiologists who are adept in PFO closure devices can prevent multi‐organ dysfunction and be lifesaving. As seen in this case, bowel ischemia can develop quickly, so rapid intervention with correction of right to left shunting as soon as safely possible should be attempted.

## Author Contributions

**Jack Franke:** data curation, writing – review and editing. **Christopher Koo:** conceptualization, data curation, investigation, writing – original draft. **John D. Serfas:** investigation, visualization, writing – original draft. **Matea Malinovic:** data curation, investigation, writing – review and editing. **John Ashcraft:** investigation, writing – review and editing. **Brigid C. Flynn:** investigation, writing – review and editing.

## Funding

The authors have nothing to report.

## Consent

Patient consent was given by the patient for writing and publishing this case report.

## Conflicts of Interest

The authors declare no conflicts of interest.

## Data Availability

Data sharing not applicable to this article as no datasets were generated or analyzed during the current study.
